# Delivering medical leadership training through the Healthcare Leadership Academy: a four year analysis

**DOI:** 10.1186/s12909-024-05031-y

**Published:** 2024-02-25

**Authors:** Azmaeen Zarif, Soham Bandyopadhyay, George Miller, Johann Malawana

**Affiliations:** 1 School of Clinical Medicine, University of Cambridge, Addenbrooke’s Hospital, Cambridge, CB2 0SP UK; 2 The Healthcare Leadership Academy, London, UK; 3 Oxford University Global Surgery Group, University of Oxford, Oxford, UK; 4 Clinical Neurosciences, Clinical & Experimental Sciences, Faculty of Medicine, University of Southampton, Southampton, Hampshire UK; 5 Wessex Neurological Centre, University Hospital Southampton NHS Foundation Trust, Southampton, UK; 6 School of Medicine, University of Central Lancashire, Preston, UK

**Keywords:** Healthcare Leadership Academy, Leadership, Networking, Healthcare, Quantitative, Qualitative, Career, Medical education

## Abstract

**Background:**

Formal leadership training is typically targeted at senior health professionals. The Healthcare Leadership Academy (HLA) was formed in 2016 to provide a leadership programme for students and early-career health professionals. This study analyses the effectiveness of the HLA scholarship programme as an intervention for improving interest in and preparing scholars for future leadership roles.

**Methods:**

Survey data was used to assess the effectiveness of the HLA Scholarship program in cultivating leadership development. Questions required either multiple-choice, free text, ranking or Likert scale (‘strongly agree’, ‘agree’, ‘neither agree nor disagree’, ‘disagree’, ‘strongly disagree) responses. Participants spanned six regions (London, Newcastle, Bristol, Belfast, Edinburgh, and Amsterdam) in four countries (England, Scotland, Northern Ireland, and the Netherlands). Descriptive statistical analyses were conducted, and insights were drawn from the open-ended survey questions using a leadership framework.

**Results:**

Seventy participants who underwent the course between 2016 and 2020 completed the questionnaire. Nearly all (99%) found that the training provided on the programme had equipped them to be more effective leaders, with 86% of respondents stating that they were more likely to take on leadership roles. Nearly all (97.1%) found the course to be either of good or very good quality. Nineteen insights were identified from free text responses that fitted under one of the four themes of the leadership framework: “optimising”, “resolving uncertainty”, “enhancing adaptability”, and “promulgating a vision”.

**Conclusions:**

Healthcare leadership is a non-negotiable component of healthcare delivery in the 21st Century. As healthcare professionals, it is our duty to be effective leaders confident and competent in navigating the increasingly complex systems within which we operate for the benefit of ourselves, colleagues, and patients. By accounting for known shortcomings and developing ameliorative measures, the HLA Scholarship programme addresses unmet needs in a structured manner to support effective long-term healthcare leadership development.

**Supplementary Information:**

The online version contains supplementary material available at 10.1186/s12909-024-05031-y.

## Background

Good healthcare leadership is vital for delivering high-quality, patient-centred healthcare [1, 2]. Leadership is distinct from management. Managers (derived from the Latin word *manus* meaning hand) work with people in roles they have been given to handle the system and ensure things are done right. Leaders form interpersonal relationships with people and influence their actions or behaviours using these relationships. Many managers will be leaders. As per Goleman’s model, participative management involves managers using relationships with people to persuade them on a course of action wherever possible [3]. However, managers can fall back on resource and position power when necessary, and often use position power when nothing else is available [4, 5]. On the other hand, leadership is not restricted to those in positions of power. Leaders are found in every part of an organisation. They are people with moral and intellectual abilities to visualise courses of actions that are best for the organisation, and can influence people to achieve that vision [6]. Therefore, leaders do not need to be managers. Managers tend to rely on the harder Ss of the 7-S framework: strategy, structure, and systems. Leaders tend to operate on the soft Ss of style, staff, skills and shared beliefs [7].

Leadership in healthcare is often considered to be distinct from leadership in other industries due to the unique challenges and complexities of the healthcare system. Healthcare leaders are responsible for managing a wide range of resources, including medical personnel, equipment, and facilities, as well as coordinating the delivery of care to patients. To effectively manage these resources and direct efforts, healthcare leaders need to have a deep understanding of the medical field and the specific needs of their patients and community. This often requires specialised medical knowledge and training, as well as the ability to understand and navigate the complex regulations and policies that govern healthcare systems. Healthcare professionals from all backgrounds have expertise in this area; however there is a paucity of research on leadership within the clinical environment among healthcare staff other than doctors.

Therefore, it is imperative that the evidence base for effective leadership within healthcare professionals is developed. Evidence from the wider literature suggests that training is required to produce effective leaders. Leadership training and development programs have been shown to improve the skills and knowledge of leaders, which in turn lead to better performance and outcomes [8]. In fact, leadership training programs that were tailored to the specific needs of the individuals participating in the program were most effective in improving a leader’s skills [9]. All healthcare professionals are required to demonstrate and develop their leadership skills throughout the course of their career with many of the UK Royal Colleges revising their specialty curricula to emphasise the need for competency in leadership. Medical schools are also required to incorporate leadership training under the GMC’s “Generic professional capabilities framework” [10]. Meeting a requirement is not, however, the same as providing adequate training. Formal leadership training has been sparse and typically targeted at senior health professionals [11]. In addition, the offering of leadership development courses lack long-term outcomes or a standard framework for evaluation [12]. There is therefore a clear and compelling need for leadership training at all stages of a healthcare professional's careers, so that healthcare professionals can identify and assume leadership roles for the potential betterment of patient outcomes.

To this extent, the Healthcare Leadership Academy (HLA) was formed in 2016 in response to demand from early career health professionals and students to learn about leadership. The one-year HLA scholarship is a prestigious scholarship that nurtures the next generation of leaders by systematically identifying, developing, and supporting those with the capability and ambition to take on leadership roles locally, nationally, and internationally. The scholarship process aims to develop participants’ understanding of leadership by bridging theory with practice, providing opportunities for participants (with assessment and feedback) to apply theory in sufficiently-challenging environments, exposing participants to different perspectives, and creating long-term follow-up within communities to ensure ongoing development [6, 13–15].

We conducted this study to analyse the effectiveness of the scholarship program – judged across various self-assessed metrics – on the role of the HLA in improving interest in and effectively preparing healthcare professionals and students for future leadership involvement.

## Methods

### The Healthcare Leadership Academy scholarship programme

To date, over 500 scholars have been selected across 37 cohorts spanning six regions (London, Newcastle, Bristol, Belfast, Edinburgh, and Amsterdam) in four countries (England, Scotland, Northern Ireland, and the Netherlands). The programme is open to applications from healthcare professional students and registered healthcare professionals. The application process consists of two phases. Applicants submit a personal statement and video introduction, describing their leadership experience and motivation to apply for the programme. If shortlisted, they are then interviewed by the Founder of the HLA (JM) before the final selection is made. Application numbers and success rates are shown in Table 1.

**Table 1:** HLA Scholarship Application Statistics 2016–2022. Waitlist refers to the number of individuals who started the application process each year by signing up to the application portal. Those who jointed the waitlist received the first invite to apply in the next cohort. The waitlist system commenced in 2019 for the 2020 cohort. The success rate is defined as the percentage of applicants receiving offers

Year	Waitlist	Applications submitted	Shortlisted for interview (N)	Shortlisted for interview (%)	Offers	Acceptance rate (%)
2016	-	27	27	100%	12	44%
2017	-	44	44	100%	14	32%
2018	-	72	45	63%	47	65%
2019	-	152	120	79%	106	70%
2020	621	180	122	68%	105	58%
2021	1821	274	201	73%	166	61%
2022	912	104	93	89%	79	76%

The programme utilises a combination of online resources (webinars, online content, and assignments), in-person sessions and mentoring from established leaders. HLA Scholars are tasked with leading both a project and a campaign over the course of 12 months on this course, and are supported through the process. Each scholar is guaranteed to have at least 7 contact days (10 am to 4 pm) with the HLA cohort directors. These contact days are spread across the year, and vary between cohorts of scholars. The core contacts days aims to develop leadership skills across six tenets: communication, negotiation, management, teamwork, philosophy, and innovation and entrepreneurship.

The programme is formally supported by Medics.Academy, Health Education England North East, Health Education England South West, and the Council of Deans of Health. It is mapped against the Masters-level programme that Medics.Academy runs in partnership with the School of Medicine at the University of Central Lancashire (UCLan) and Scholars may partially complete credit requirements based upon the completion of the programme.

### Study design

We conducted a study using a survey developed to collect data on the effectiveness of the HLA Scholarship program in cultivating leadership development. Scholars who took part in the first four cohorts of the HLA Scholarship programme (2016–17, 2017–18, 2018–19, and 2019–20; *n* = 179) were contacted via email with a request to complete the 22-question survey (see Supplementary Materials for a copy). Scholars from 2020–21, 2021–22, and 2022–23 cohorts had amended activities due to the COVID-19 pandemic and were therefore excluded from the analysis of the survey results due to activities now returning to how they were pre-pandemic. The questions required either multiple-choice, free-text, ranking (between 1 to 12) or Likert scale (‘strongly agree’, ‘agree’, ‘neither agree nor disagree’, ‘disagree’, ‘strongly disagree) responses. Two independent colleagues who were identified due to their expertise in healthcare leadership, unrelated to the HLA, checked the survey’s reliability based on the six-step method [16]. The survey was found to have face validity, criterion validity, construct validity, and content validity.

### Data collection

All surveys were conducted using the Survey Monkey platform. The link was circulated via email. Scholars who had not completed the survey were regularly followed up to maximise the response rate (four total follow-up emails sent weekly over the course of a month). Surveys were anonymised to minimise response bias; we were only able to view the results if scholars completed all sections, with all participants providing informed written consent for their data to be used in research.

### Framework

To structure our qualitative data analysis, we employed the framework outlined by Parry, which emphasises four aspects of leadership: optimising, resolving uncertainty, enhancing adaptability, and promulgating a vision [17]. Optimising refers to the ability of leaders to make the best of situations, use resources optimally, and aim for excellence. Resolving uncertainty is the processes through which leaders help in clarifying ambiguities for both themselves and their followers, and enhancing adaptability is how leaders promote adaptability among followers and within themselves. Furthermore, Parry clarifies the role of effective leaders in promulgating a vision: defining, communicating a clear vision, and helping followers navigate change situations.

### Data analysis

Descriptive statistical analyses were performed using RStudio (Version 1.2.5042) to summarise the quantitative data from the student survey instrument.

The open-ended responses from the survey data were initially reviewed to understand the breadth and depth of the content. Identifiers were removed from transcripts to preserve anonymity. Responses were then coded based on Parry's four aspects. Two investigators (AZ and SB) independently reviewed all responses from the completed surveys [18]. While coding, attention was given to emergent insights that could fall within Parry's framework, capturing nuances that might provide additional insights into leadership practices [18, 19]. The other co-authors (GM and JM) then reviewed the emergent insights and exemplar quotations. All authors contributed to the development of the insights from the open-ended survey questions.

## Results

**Fig. 1:**
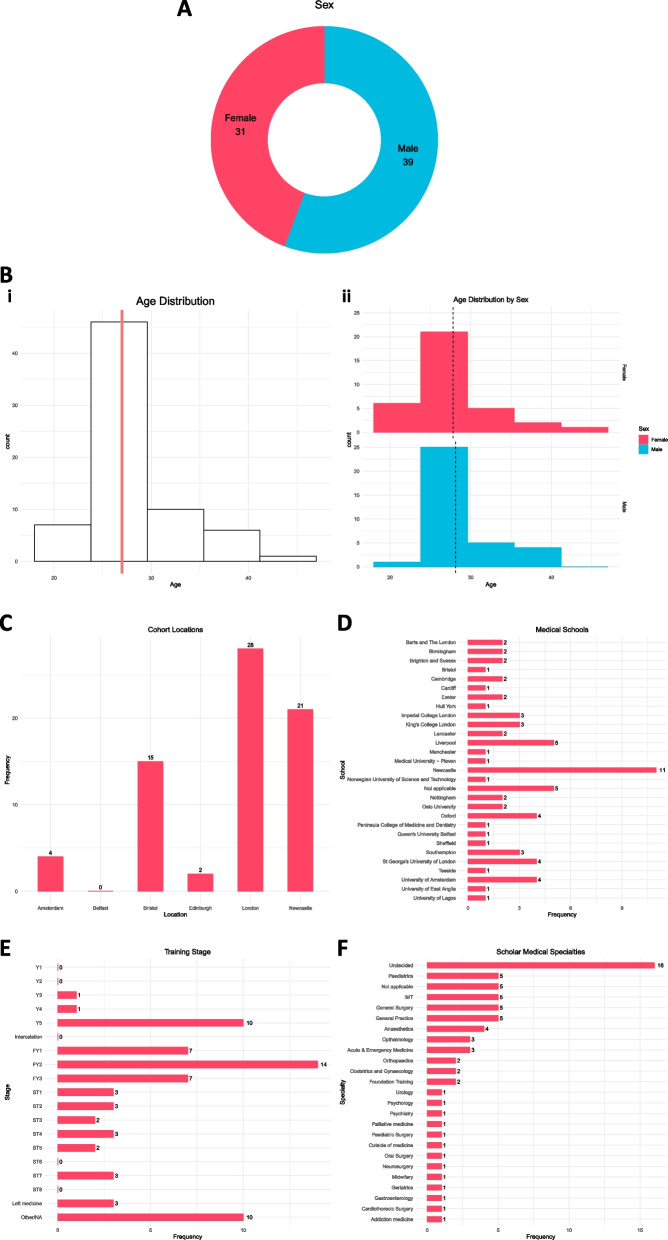
Cohort demographics. **A** Sex distribution. **B** Age distribution, including by sex. Line indicates median age. **C** Collated cohort locations. **D** Medical schools (where applicable) where scholars were either currently being trained or were trained at during their scholarship year. **E** Training stage (FY: Foundation Year; ST: Specialist Training) with training grades only applicable for doctors. **F** Distribution of respective specialties, where appropriate, for trainees

We had a questionnaire completion rate of 39.1% (*n* = 70), of which 44.3% of respondents were female (Fig. 1A). The median age of respondents was 25 (Fig. 1B; males had a median age of 25 and females had a median age of 32). The majority of respondents had been part of the London (40.0%) or Newcastle (30.0%) cohorts, which were two of the first to be launched (Fig. 1C). Scholars had been trained at various UK and international medical schools (Fig. 1D) and the majority were practising trainee doctors below the consultant grade (FY1-ST8) (Fig. 1E). There were a wide range of specialty trainees, although many were undecided as of the time of the questionnaire (Fig. 1F).

### The HLA scholarship programme promotes future leadership involvement

**Fig. 2:**
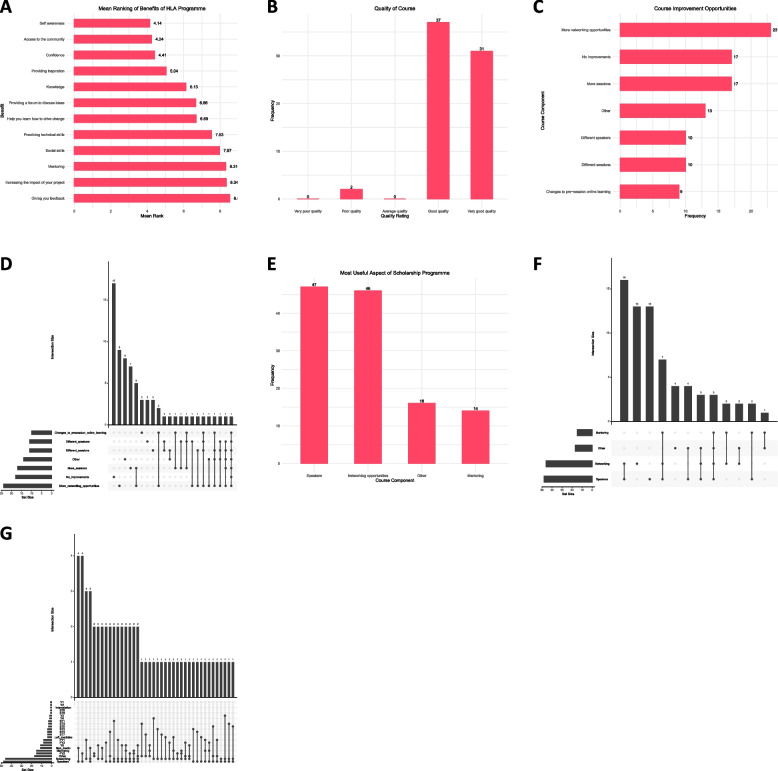
HLA scholarship programme course evaluation. **A** Average ranking of the benefits of the HLA scholarship programme (1 = highest, 12 = lowest. **B** Course quality responses. **C** Areas of course improvement feedback. **D** Upset plot to identify any consistently-identified areas of improvement of the course. **E** Most useful aspect of the course from selected major domains of mentoring, networking, and speakers. **F** Upset plot indicating overlap of domains most valued by participants. **G** Upset plot outlining useful areas of course valued based on training stage

When scholars were asked to evaluate the overall course, the vast majority found it to be an overwhelmingly positive experience. Nearly all (99%) found that the training provided on the programme had equipped them to be more effective leaders, with 86% of respondents stating that they were more likely to take on leadership roles. Mean ranking of the benefits of the programme found that among the range of components on offer, scholars valued how the programme improved their self-awareness and confidence; in addition, other high-ranking benefits included the networking component and an improved knowledge of how to navigate healthcare systems and problems for effective outcomes in order to drive change (Fig. 2A). Nearly all (97.1%) found the course to be either of good or very good quality (Fig. 2B). However, there are suggested areas of improvement (Fig. 2C), which programme directors aim to incorporate for future cohorts. It must be noted that there were no overlaps between areas of improvement to suggest that any one area consistently lagged, in other words, different scholars had different needs they requested for further improvement (Fig. 2D). Of the major areas identified for a successful programme as mentioned previously, we found that scholars also valued the components of mentorship, networking, and speakers (in contrast to the other components on offer) (Fig. 2E). Networking and speakers as the primary draw (Fig. 2F) were particularly highlighted by scholars earlier in their training (Fig. 2G). The vast majority (87%) either agreed or strongly agreed that HLA covered the gap of the hidden curriculum that their respective institutions had not delivered. We defined the hidden curriculum as important skills necessary for a job in healthcare which are not formally taught either as part of the official curriculum nor as a trainee. The majority (80%) further agreed or strongly agreed that there is a need for more leadership training in the early stages of one’s career (medical school or foundation training).

### Insights from open-ended survey questions

Nineteen insights were identified that fell within Parry’s framework. A summary of these insights are presented in Table 2.

**Table 2:** Insights from open-ended survey questions categorised by Parry’s framework

Framework themes	Insights
Optimising﻿	Knowledge gained
	Development of soft skills
	Engaging content about leaders excelling
Resolving Uncertainty	Understanding of own priorities
	Accountability mattering
Enhancing Adaptability	Ability to foster connections
	Greater range of views considered
Promulgating a Vision	Transitioning into leadership roles
	Collaboration over competition

#### Optimising

Central to the program’s success was its ability to enhance and optimise leadership skills among scholars. These skills, encompassing effective communication, problem-solving, and decision-making, were not just about managing tasks but about excelling in them. Scholars noted that the development of their soft skills, such as emotional intelligence and strategic communication, was crucial in optimizing team dynamics and achieving excellence. This focus on soft skills was unique to the program and highly valued, as it addressed areas often overlooked in other leadership courses.

*‘The HLA taught me the fundamental principles of leadership, helped boost my confidence and overall skillset’*

*Participant 12314573348*

*“aspects of the course that was unique compared to other leadership courses was the session on media and the other on personalities. Those two sessions really impacted how I portray myself internally and externally.”*

*Participant 12657080771*

*‘I had taken up previous leadership training programmes in the past, but The HLA was pivotal in being very accessible (whilst still a competitive entry)’*

*Participant 12249091931*

Feedback on the course’s structure was generally positive, with suggestions for optimising it further by involving speakers in more influential positions. Nevertheless, the current speakers, who demonstrated how to achieve excellence with limited resources, were well-received. The course's practical components, including leading projects, were seen as valuable opportunities for scholars to apply and refine their skills in real-world scenarios. However, it was acknowledged that some skills require ongoing development beyond the confines of a single project.

In addition, a few scholars referenced that the earlier projects offered by the HLA could feel like they were operationalising parts of the organisation itself; however, this sentiment reduced in pervasiveness since the introduction of the opportunity for individuals to conduct projects independently of the HLA. This evolution reflects a commitment to optimising the learning experience, ensuring that scholars are equipped not only with leadership skills but also with the ability to make the best of any situation they encounter.

#### Resolving uncertainty

Scholars reported that by undertaking the HLA course, they were able to better understand their own goals and priorities, and to make more deliberate choices about how to use their time and talents to make a meaningful impact in their work. Scholars believed the HLA course played a pivotal role in resolving uncertainties regarding their leadership paths.

*“Above all the HLA has focused me into the mindset of “what do I want to achieve”. Before this course I had very diffuse ideas about getting into leadership to make change, now I feel more able to define my objectives so that I can apply to roles based on how I actually think that I can make a useful change in my work.”*

*Participant 12219639873*

After completing the course, taking leadership roles without the ability to be accountable for them was deemed inappropriate by scholars, and scholars appreciated the importance of “saying no” to roles when circumstances meant that the individual would not be able to fulfil the role to the best of their ability. This understanding helped to resolve an uncertainty often faced by leaders about when to take on or decline opportunities. Additionally, scholars recognised that accountability extended to their communication, both written and oral, meaning that an individual should be comfortable with anything they say being attributed to their name. There was recognition that leaders are held to a high standard of integrity and professionalism. This resolved scholars’ uncertainties over how to approach various issues, as they realised that their actions always need to align with the values and goals of their organisation.

*‘I remember one of our first workshop sessions, and somebody asked why we write our names on feedback forms. Johann said something along the lines of “learning to stand by your words no matter how they’re perceived”. I’ll never forget that – it’s incredibly useful for all aspects of life.’*

*Participant 12314573348*

#### Enhancing adaptability

Scholars found that engaging with peers at similar stages in their leadership journey, coupled with mentorship tailored to their specific ambitions, contributed to their ability to adapt to take on their leadership roles. Scholars reported that their self-confidence grew, and they felt more able to reach out to colleagues to achieve a goal. In addition to learning from the course material and the cohort directors, scholars recognised the value of having speakers with a diversity of backgrounds as well being part of a community of scholars with their own experiences of leadership. The diversity in viewpoints, backgrounds, and pathways was reported to be motivating to scholars. Scholars noted that the diversity among speakers and the cohort itself served as a catalyst for developing adaptability. It motivated them to consider multiple approaches to managing situations and leading projects, thereby fostering a flexible and adaptable mindset.

*‘I really enjoyed the opportunity to become part of the HLA community and to learn from the in-person sessions which were very engaging, interesting and were from a broad array of topics that covered many areas that I wouldn’t have looked into myself.’*

*Participant 12219639873*

#### Promulgating a vision

Participants who completed the course reported significant growth in their experience and confidence, which translated into successful transitions to leadership roles at various levels—local, regional, national, and international. This success was attributed not only to individual achievement but also to the ability to share and collaborate on a unified vision of leadership.

*‘definitely helped me to understand…the opportunities that collaboration can unlock both for the group and on a personal level.’*

*Participant 12219639873*

An important realisation among the scholars was that leadership success does not stem from competition, but rather from collaboration. This understanding was reinforced through negotiation workshops within the course, which emphasized the value of working together towards a common vision. These exercises were not just about building skills; they were about shaping a mindset where leaders see the value in bringing people together, aligning their efforts towards shared goals, and effectively navigating change situations.

### Long term leadership perceptions

**Fig. 3:**
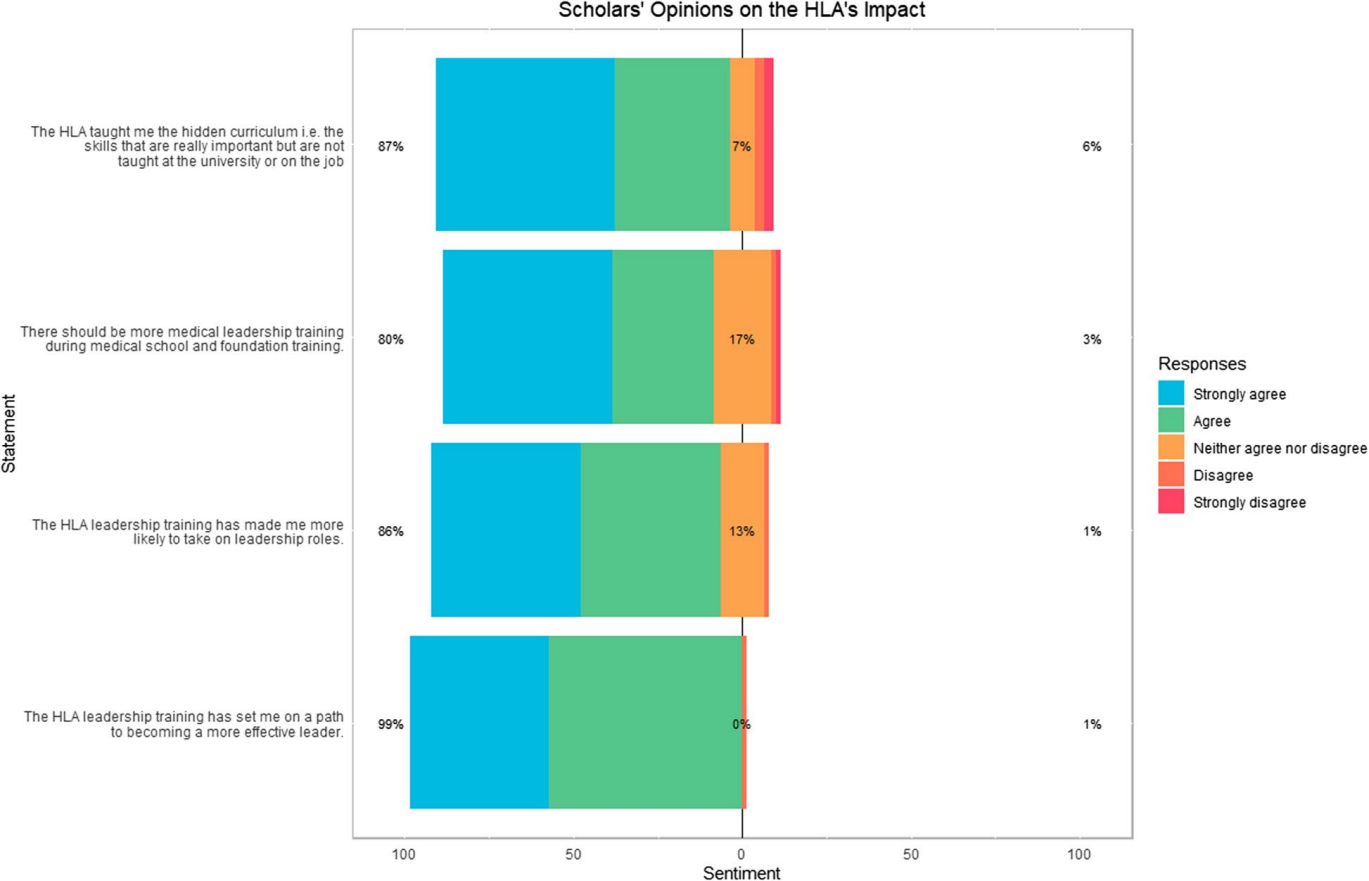
Likert scale responses outlining scholars’ opinions of the impact of the HLA programme on their personal development with respect becoming an effective healthcare leader

**Fig. 4:**
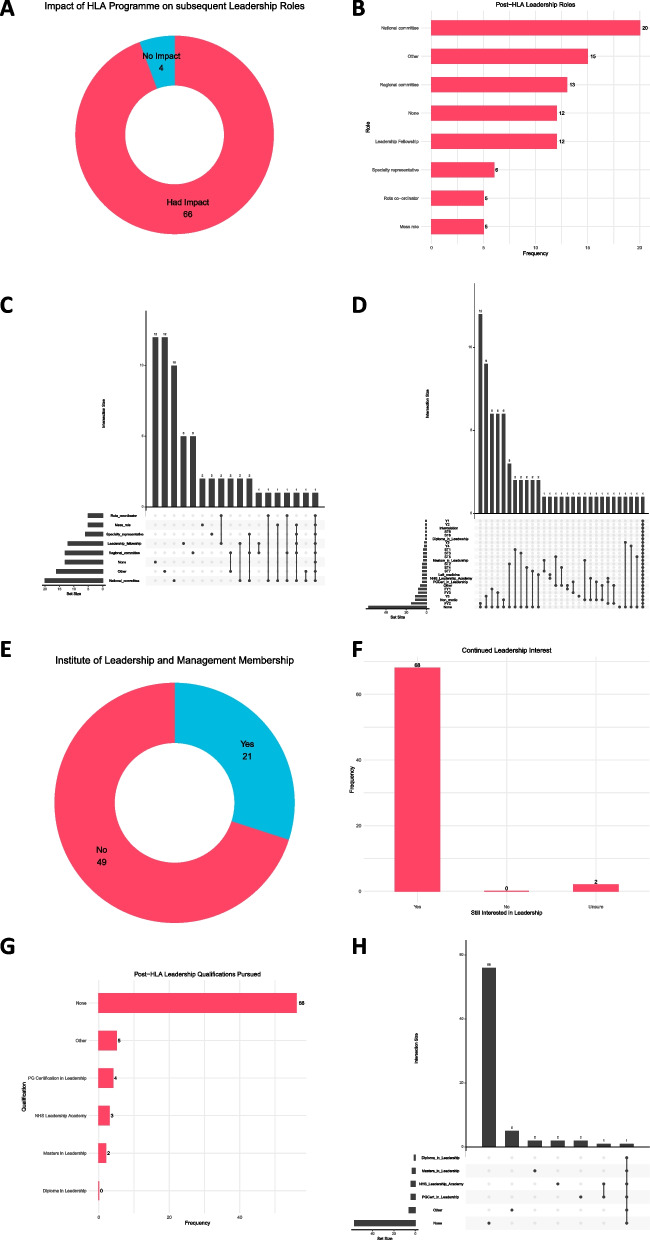
Assessing the long-run impact of the HLA on subsequent leadership-related activities undertaken by scholars after their scholarship year. **A** Scholars’ opinions on the influence of the HLA on their pursual of subsequent leadership roles. **B** Breakdown of leadership roles pursued by scholars after their HLA year. **C** Upset plot illustrating overlaps of roles pursued by individual scholars. **D** Upset plot indicating interaction between leadership roles pursued and seniority (training stage). **E** Breakdown of proportion of scholars who became members of the Institute of Leadership and Management after completing the scholarship programme (equivalent level of qualification required for membership) for continued professional development purposes. **F** Continued leadership interest after completing scholarship year. **G** Leadership-related qualifications pursued after completion of scholarship year. **H** Upset plot illustrating overlap between common leadership qualifications pursued

We aimed to determine if these positive attitudes had translated into tangible outcomes. Corroborating the positive attitudes towards pursuing future leadership roles (Fig. 3), the vast majority (94.2%) attributed the HLA programme to have influenced subsequent roles they had undertaken (Fig. 4A). Scholars were found to have occupied leadership roles at various levels ranging from local organisations through to regional and national committees (Fig. 4B). The majority of scholars did not pursue multiple roles (Fig. 4C) and this trend for being more discriminating about their future leadership persisted even after accounting for training stage (Fig. 4D), but did not reach significance. Completion of the programme is deemed to sufficiently qualify scholars for membership (and now following further additions, fellowship) of the Institute of Leadership and Management for continued professional development. We found that 30% of scholars had taken up the option to become members on this basis (Fig. 4E). Whilst nearly all (97.1%) of scholars continued to retain an interest in healthcare leadership (Fig. 4F), 80% of respondents had not pursued further leadership qualifications (Fig. 4G) after completing the scholarship programme, with only one scholar having pursued multiple qualifications (Fig. 4H).

## Discussion

### Principal findings

The HLA Scholarship programme supports the leadership development and ambitions of scholars. Those who took part in the programme, across all four cohorts studied, largely found it to be a positive experience. It addresses gaps in their training and equips them with the necessary knowledge, confidence, and experience, through various components including, but not limited to, speakers, a network of like-minded individuals, and mentoring to directly contribute to realising future leadership aspirations. Over the years, the programme has developed in its curriculum and offerings and, by accounting for feedback received from past scholars, potential areas of further improvement have been identified.

### Implication of findings

Leadership is a central component of consistently high-quality care. In the UK, there is a growing emphasis for doctors and healthcare workers as leaders within the National Health Service (NHS). This has been codified in recent updates including *A High Quality Workforce: NHS Next Stage Review,* which establishes a triad for the role of the doctor with leadership as a central tenet; the Medical Leadership Competency Framework (MLCF), published jointly by the Academy of Medical Royal Colleges (AoMRC) with the NHS institute for Innovation and Improvement to comprehensively detail the necessary components of medical leadership; and the creations of the National leadership Council and NHS Leadership Academy to support skill development. In other words, it is evident that the future organisational development of the NHS visualises healthcare leadership as a central foundation.

Whilst efforts to address past shortcomings are admirable, a failure to address obstacles may prevent the ambitious proposals being realised. Nearly a decade has passed since the Francis Enquiry called for a review into obstacles faced by clinicians aiming to transition into leadership roles [20]. Long-standing obstacles reported include the common perception amongst doctors that do not see themselves as leaders nor a need for leadership as a central component for patient care attributing the nature of medical training which emphasis the role of the collective [21]. Other shortcomings include a lack of senior support, insufficient time allocation during training, and prohibitive personal and financial costs associated with current formal courses offered by the NHS Leadership Academy such as the Nye Bevan Programme and the 2025 Leaders Programme reaching near £4,500 and £11,000 in fees, respectively [22, 23]. The final issue to contend with is how leadership competencies should be integrated as part of clinical training. It might be reductive to conceptualise leadership skills, especially the “softer” skills of communication, delegation, and adaptability (to name a few), can be assessed in the same manner as clinical competencies. In other words, leadership is not amenable to simple checklists. At the same time, leaving individuals to chart their own pathways through extracurriculars without providing an appropriate platform fails to develop a comprehensive foundation for personal development. It is well-documented that poor clinical leadership is associated with poor patient outcomes and, in contrast, increased emphasis on clinical leadership translates to greater focus on improved patient care [1, 24, 25]. Given that leadership is a complex, multicomponent advanced competency rather than a fixed personality trait, it is a dynamic skill that can be developed through appropriate interventions [26].

Our findings suggest that a dedicated programme can be tailored to address shortcomings of current provisions for effective leadership development. The core curriculum of the scholarship programme aims to develop leadership skills across six tenets: communication, negotiation, management, teamwork, philosophy, and innovation and entrepreneurship. In doing so, it aims to address perception issues of leadership as an unknown quantity and provides a formalised structure to help participants understand and develop the skills in relevant domains. By better understanding the healthcare systems in which they operate and appreciating how to navigate it through communication and negotiations for example, scholars can better understand the mechanisms required to advocate for their patients, colleagues, and themselves. The lack of senior support is addressed via both a formal mentorship scheme as well as informal opportunities provided by the networking events, allowing individuals with similar interests to meet and form deeper connections. On the issue of cost, as a scholarship programme, it is free of charge for scholars to participate. Indeed, each scholar instead is provided with over £3000 worth of training at no cost to themselves. With regards to assessment, the programme conceptualises development as a continual process. Scholars are required to complete a scholarship project over the course of the programme. This necessitates establishing objectives and aims they hope to achieve and forming a plan that is checked by their project supervisor. There are regular check-ins throughout the year to assess how projects are progressing. The goal is to ensure said projects develop critical skills necessary for a good leader. As a result, whilst there is no formal summative assessment, the ongoing process of developing, executing, and evaluating the project over the course of the year is in itself a form of providing a structured approach for interested individuals to gauge their competencies, identify weaknesses and try solutions to overall develop their leadership profile with the support of fellow scholars, mentors, and supervisors.

Our findings support prior research into healthcare leadership development. Formalised leadership teaching has been demonstrated to improve self-confidence, teamwork, and increased self-reported ability to delegate and direct groups, all findings that our scholars have also reported experiencing improvements in [27]. Indeed, our satisfaction findings corroborate that of other groups that formalised teaching through a course, as opposed to ad-hoc provisions, improve engagement [28]. The structure has been found to help improve understanding of the systems within which they operate and how they can influence it, a particular emphasis of our course to improve confidence in advocating for others [29]. Moreover, in keeping with the key qualities that students have previously identified to be central for leadership (communication, conflict resolution, time management, negotiation, delegation, teamwork, and community service), our programme, through its core curriculum, speakers, mentorship, networking, and scholarship project component, has been designed to develop said qualities over the course of the scholarship year [30, 31]. It does so in a structured manner which has been well-received as per our results, thereby addressing the current gap or, where present, poor provisions, aiming to meet the significant international demand for greater emphasis on early-career medical leadership training [30–32]. On the issue of early-career training, it is important to highlight that the vast majority of our participants were either students or just starting their healthcare careers. We postulate that targeting individuals earlier in their career may be more beneficial to facilitate a longer time period for leadership development and outcomes. By addressing attitudes early and by providing the necessary tools to allow for effective leaders to start building their foundations from the onset, it may help healthcare professionals better recognise how to navigate the wider system for their own benefit and that of their patients. There are various postgraduate leadership programmes which are more specialty-specific [33]. However, positioning it as postgraduate training counteracts our proposal that leadership must be viewed as a core skill as a healthcare professional, one that requires the same level of continual development as clinical skills in parallel rather than an optional extra at the latter stages of one’s training. Specialty-specific programmes also lack our multidisciplinary emphasis on networking and mentoring to address broader healthcare challenges – the domains of leadership development and application need not be restricted.

### Limitations

Our study had several limitations. We had a low completion rate (39.1%), however, it is in line with meta-analytic findings of expected response rates for web-based surveys with email follow-ups [34–36]. Whilst this limited the generalisability of our quantitative analysis, our qualitative data was not dependent on sample size. Another potential limitation is the potential bias due to a self-selecting cohort skewing the data. Yet, we account for this by referring to the previously-mentioned findings that an overwhelming majority of students and healthcare professionals express the importance of and desire for healthcare leadership training. Moreover, the questions aimed to assess development as opposed to interest in leadership development. Each scholar was their own control from baseline pre-programme. Whilst we appreciate that self-reported measures may not be the most reliable measure, in this instance we would argue that it was the most appropriate. The aim was not to train to a predetermined standard but rather to establish a foundation personalised to the individual by equipping them with the necessary skills and tools for them to pursue future leadership opportunities. Both the quantitative and qualitative responses suggest that for the vast majority, this aim was fulfilled. However, a further limitation was that the survey was unable to gauge whether participants had gained about the knowledge of the core tenets of leadership during their time on the course.

There is scope for future work through cohort studies integrating expanded evaluation to measure objective changes in domains such as local team behaviours, personal clinical outcomes, and patient-reported satisfaction scores. Applying the framework and integrating into current school curricula further provides the opportunity to study changes in student attitudes and behaviours pre-programme and post-programme, with the opportunity for long-term follow-up. As the HLA continues its scholarship offering internationally, we are interested in exploring the impacts of the new modules we are developing and assessing the validity of region-specific tailored courses for more specialised training.

## Conclusion

Healthcare leadership is a non-negotiable component of healthcare delivery in the 21st Century. As healthcare professionals, it is our duty to be effective leaders confident and competent in navigating the increasingly-complex systems within which we operate for the benefit of ourselves, colleagues, and patients. By accounting for known shortcomings and developing ameliorative measures, the HLA Scholarship programme addresses unmet needs in a structured manner to support effective long-term healthcare leadership development.

### Supplementary Information

**Additional file 1. **

## Data Availability

The datasets used and/or analysed during the current study are available from the corresponding author on reasonable request.
