# Supplementary material for: Delivering medical leadership training through the Healthcare Leadership Academy: a four year analysis
Source: BMC Med Educ. 2024 Feb 25;24:194. doi: 10.1186/s12909-024-05031-y (PMC10895908; doi:10.1186/s12909-024-05031-y)
Supplement: Supplementary file 1 — Additional file 1. [file 12909_2024_5031_MOESM1_ESM.pdf]

Supplementary Materials

S1. Delivering Medical Leadership Training Through The Healthcare Leadership Academy - Questionnaire

1. Please enter your name (This will be used only to confirm who is yet to complete the survey. Your answers below will be anonymised and not in any way linked to your name).

2. I consent for this data to be analysed and published. Data will be anonymised and there will be no individual identifying information shared or published – tick for consent

3. Has completing the course had an impact on your subsequent leadership roles? Please select Yes/No and answer why on the comment box.

4. What did you think of the quality of the course? (Very poor, Poor, Average, Good quality, Very good quality). Why did you pick the above? (i.e. what was good/bad)

5. What was the most useful aspect of this course? (Mentoring, Speakers, Networking opportunities, Others [please specify in comment box])

6. Are you still interested in leadership? (Yes, No, Unsure)

7. To what extent do you agree with this statement: The HLA taught me the hidden curriculum i.e. the skills that are really important but are not taught at the university or on the job. (Strongly disagree, Disagree, Neutral, Agree, Strongly Agree). Why did you pick the above option? (please give further details).

8. How could the course have been improved? (More sessions, More networking opportunities, Changes to pre-session online learning, Different speakers, Different sessions, No improvements, Other [please specify in comment box])

9. Have you taken on a leadership role post-HLA? (tick all that apply: Leadership, Rota co-ordinator, Mess role, Specialty representative, Regional committee, National committee, No, Other). If yes or other, please give more detail on the role in the comment box.

10. What speciality have you chosen to go into?

11. Have you gained a subsequent leadership qualification? (tick all that apply: NHS Leadership Academy, Masters in Leadership, Diploma in Leadership, PG Certification in Leadership, No, Other). If yes or other - please give further details on the qualification(s) if applicable.

12. Are you a member of the Faculty of Medical Leadership and Management (FMLM)? (Yes, No)

13. To what extent do you agree with this statement: The HLA leadership training has set me on a path to becoming a more effective leader. (Strongly disagree, Disagree, Neutral, Agree, Strongly Agree). Why did you pick the above option? (please give further details).

14. To what extent do you agree with this statement: The HLA leadership training has made me more likely to take on leadership roles. (Strongly disagree, Disagree, Neutral, Agree, Strongly Agree). Why did you pick the above option? (please give further details).

15. To what extent do you agree with this statement: There should be more medical leadership training during medical school and foundation training. (Strongly disagree, Disagree, Neutral, Agree, Strongly Agree). Why did you pick the above option? (please give further details).

16. In what way has the HLA benefited you? Rank all 12 options (Confidence, Self awareness, Knowledge, Practicing technical skills, Access to the community, Mentoring, Providing inspiration, Social skills, Increasing the impact of your project, Giving you feedback, Help you learn how to drive change, Providing a forum to discuss ideas) from greatest benefit =1, to least benefit =12.

17. What is your gender?

18. What is your age?

19. What stage of your training are you in?

20. What cohort were/are you in? (1, 2, 3, 4, 5, 6, 7, 8, 9, 10)

21. What is/was your cohort location? (London, Amsterdam, Newcastle, Belfast, Bristol, Edinburgh)

22. Which medical school did you go to?
